# Antibiotic resistance and biofilm production among the strains of *Staphylococcus aureus* isolated from pus/wound swab samples in a tertiary care hospital in Nepal

**DOI:** 10.1186/s12941-017-0194-0

**Published:** 2017-03-23

**Authors:** Ankit Belbase, Narayan Dutt Pant, Krishus Nepal, Bibhusan Neupane, Rikesh Baidhya, Reena Baidya, Binod Lekhak

**Affiliations:** 1 Department of Microbiology, GoldenGate International College, Battisputali, Kathmandu, Nepal; 2 Department of Microbiology, Grande International Hospital, Dhapasi, Kathmandu, Nepal; 3 Department of Pathology, B&B Hospital, Gwarko, Lalitpur, Nepal; 4 Central Department of Microbiology, Tribhuvan University, Kirtipur, Nepal

**Keywords:** *S. aureus*, Inducible clindamycin resistance, Biofilm, MRSA, Linezolid, Vancomycin

## Abstract

**Background:**

The increasing drug resistance along with inducible clindamycin resistance, methicillin resistance and biofilm production among the strains of *Staphylococcus aureus* are present as the serious problems to the successful treatment of the infections caused by *S. aureus*. So, the main objectives of this study were to determine the antimicrobial susceptibility patterns along with the rates of inducible clindamycin resistance, methicillin resistance and biofilm production among the strains of *S. aureus* isolated from pus/wound swab samples.

**Methods:**

A total of 830 non-repeated pus/wound swab samples were processed using standard microbiological techniques. The colonies grown were identified on the basis of colony morphology, Gram’s stain and biochemical tests. Antimicrobial susceptibility testing was performed by Kirby–Bauer disc diffusion technique. Detection of inducible clindamycin resistance was performed by D test, while detection of methicillin resistant *S. aureus* (MRSA) was performed by determination of minimum inhibitory concentration of oxacillin by agar dilution method. Similarly, detection of biofilm formation was performed by microtiter plate method. Strains showing resistance to three or more than three different classes of antibiotics were considered multidrug resistant.

**Results:**

Total 76 samples showed the growth of *S. aureus*, among which 36 (47.4%) contained MRSA and 17 (22.4%) samples were found to have *S. aureus* showing inducible clindamycin resistance. Among the *S. aureus* isolated from outpatients, 41.9% were MRSA. Highest rates of susceptibility of *S. aureus* were seen toward linezolid (100%) and vancomycin (100%). Similarly, *S. aureus* isolated from 35 (46.1%) samples were found to be biofilm producers. Higher rate of inducible clindamycin resistance was seen among MRSA in comparison to methicillin susceptible *S. aureus* (MSSA). Similarly, higher rates of multidrug resistance and methicillin resistance were found among biofilm producing strains in comparison to biofilm non producing strains.

**Conclusions:**

The rate of isolation of MRSA from community acquired infections was found to be high in Nepal. Increased rate of inducible clindamycin resistance as compared to previous studies in Nepal was noted. So for the proper management of the infections caused by *S. aureus*, D test for the detection of inducible clindamycin resistance should be included in the routine laboratory diagnosis. Further, detection of biofilm production should also be included in the routine tests. Linezolid and vancomycin can be used for the preliminary treatment of the serious infections caused by *S. aureus*.

## Background

The increasing rates of drug resistance among *S. aureus* to commonly used antibiotics and emergence of methicillin resistant strains for which limited treatment options exist have created a great problem to the management of the infections caused by *S. aureus* [1]. The accurate local antibiotic susceptibility data along with knowledge about the local prevalence of methicillin resistance may be helpful for starting proper preliminary treatment of the infections caused by *S. aureus* [1]. The increased prevalence of drug resistance mainly methicillin resistance among the strains of *S. aureus* has impelled the usage of macrolide–lincosamide–streptogramin B (MLS_B_) antibiotics mainly clindamycin for the treatment of the infections caused by *S. aureus* [2]. However, there are reports of increasing resistance to MLS_B_ therapy particularly to clindamycin due to haphazard use of these antibiotics [2]. The inducible clindamycin resistance is responsible for treatment failure of the infections caused by *S. aureus* treated with clindamycin, as it can not be detected in the routine laboratory tests if the erythromycin and clindamycin are not kept adjacent to each other while performing antimicrobial susceptibility testing [2–4]. D test is one of the easiest methods that can be employed to detect the strains of *S. aureus* showing inducible clindamycin resistance.

Similarly, another problem with the treatment of infections caused by *S. aureus* is biofilm formation. Biofilm formation is a defense mechanism of *S. aureus* [5]. Bacteria protected by biofilms are resistant to host defense mechanisms and show resistance to standard antibiotic therapy [5].

So, in this study we determined the drug susceptibility patterns of the strains of *S. aureus* isolated from pus/wound swab samples. Further, we also studied the prevalence of inducible clindamycin resistance and methicillin resistance along with rate of biofilm production.

## Methods

A cross sectional descriptive study was conducted among the patients attending B & B hospital, Lalitpur, Nepal from March 2015 to September 2015. A total of 830 pus/wound swab samples (400 from inpatients and 430 from out patients) received were processed using standard microbiological techniques [6]. The colonies grown were identified on the basis of colony morphology, Gram’s stain, and biochemical tests [7]. Yellow colored colonies on mannitol salt agar, which were Gram positive cocci, catalase positive, slide and tube coagulase positive, hydrolysed gelatin, showed beta-hemolysis on blood agar, methyl red positive, Voges–Proskauer positive, nitrate reduction positive, fermentative, urease positive, DNase producing, lactose, mannitol, maltose, mannose, sucrose and trehalose fermenting, alkaline phosphatase positive were confirmed as *S. aureus*. Antimicrobial susceptibility testing was performed by Kirby Bauer disc diffusion technique following clinical and laboratory standards institute (CLSI) guidelines [8]. The concentration of suspension of the test organism was made equivalent to 0.5 McFarland standards. And lawn culture was performed on Mueller–Hinton agar plate. Then the antibiotic discs were placed over the lawn culture and the plate was incubated aerobically at 35 °C for 24 h. Finally the plate was observed for zone of inhibition and interpreted according to CLSI guidelines. The antibiotic discs used were penicillin-G (10 units), cefoxitin (30 μg), ciprofloxacin (5 μg), clindamycin (2 μg), chloramphenicol (30 μg), erythromycin (15 μg), gentamicin (10 μg), tetracycline (30 μg), cotrimoxazole (25 μg), vancomycin (30 μg) and linezolid (30 μg). Strains showing resistance to three or more than three different classes of antibiotics were considered multidrug resistant [9].

### Detection of methicillin resistant *S. aureus*

The strains of methicillin resistant *S. aureus* were detected by determination of minimum inhibitory concentration of oxacillin by agar dilution method [8, 10]. Different concentrations of oxacillin ranging from 0.0625 to 32 μg/ml were prepared. One milliliter of each of these concentrations was added to 19 ml of Mueller–Hinton agar cooled to around 50 °C and allowed to solidify in sterilized petri plates. Then the concentration of the broth containing test organism was adjusted to 0.5 McFarland standards. Prior to inoculation, further (1:10) dilution of the broth containing test organism was performed. The plates were then inoculated with the test organisms and were incubated aerobically at 35 °C for 24 h, which were then examined for bacterial growth and interpreted according to CLSI guidelines.

### Screening of inducible clindamycin resistance in *S. aureus*

The inducible clindamycin resistance was detected by D test [8]. Erythromycin (15 μg) disc and clindamycin (2 µg) disc were placed 15–26 mm edge to edge apart in Mueller–Hinton agar plate inoculated with the test isolate. The plates were incubated aerobically at 35 °C for 16–18 h. Flattening of the zone of inhibition of clindamycin adjacent to the erythromycin disc was regarded as D test positive.

### Screening of biofilm production among the strains of *S. aureus*

The detection of biofilm production among the strains of *S. aureus* was performed by microtiter plate method [11]. The *S. aureus* was grown in 96 welled microtiter plate containing trypticase soya broth with 2% glucose, at 35 °C for 48 h. The optical density value of the bacteria that coat the wall of the wells was determined with the help of enzyme linked immunosorbent assay reader after staining with crystal violet. The organisms were identified as biofilm producer or biofilm non producer on the basis of the observed optical density values.

For quality control S. *aureus* ATCC 25923 was used.

### Data analysis

For data analysis SPSS version 21.0 was used. Chi square test was applied and *p* value <0.05 was considered statistically significant.

## Results

Among total 830 pus/wound swab samples processed, 364 (43.9%) were culture positive. Out of which, *S. aureus* was isolated from 76 (20.9%) samples. Among the 76 *S. aureus* isolated, 45 (59.2%) were MDR, 36 (47.4%) were MRSA, 17 (22.4%) were D test positive and 35 (46.1%) were biofilm producers.

Out of 76 *S. aureus* isolated, 43 (56.6%) were isolated from outpatients, whereas 33 (43.4%) were isolated from inpatients. Among the *S. aureus* isolated from outpatients, 41.9% were MRSA, while among the *S. aureus* isolated from inpatients 54.5% were MRSA.

### Antimicrobial susceptibility patterns of *S. aureus* isolated

Among all the *S. aureus,* highest rates of susceptibility were seen toward linezolid (100%) and vancomycin (100%) followed by tetracycline (98.7%) and chloramphenicol (94.7%) (Table 1).

**Table 1:** Antimicrobial susceptibility patterns of *S. aureus* isolates

Antibiotics	MRSA (n = 36)	MSSA (n = 40)	Total (n = 76)
Penicillin	0 (0%)	2 (5%)	2 (2.6%)
Cefoxitin	0 (0%)	40 (100%)	40 (52.6%)
Ciprofloxacin	8 (22.2%)	13 (32.5%)	21 (27.6%)
Cotrimoxazole	10 (27.8%)	18 (45%)	28 (36.8%)
Gentamicin	22 (61.2%)	30 (75%)	52 (68.4%)
Chloramphenicol	35 (97.2%)	37 (92.5%)	72 (94.7%)
Erythromycin	10 (27.8%)	24 (60%)	34 (44.7%)
Clindamycin	32 (88.9%)	36 (90%)	68 (89.5%)
Tetracycline	35 (97.2%)	40 (100%)	75 (98.7%)
Vancomycin	36 (100%)	40 (100%)	76 (100%)
Linezolid	36 (100%)	40 (100%)	76 (100%)

### Inducible clindamycin resistance among strains of *S. aureus*

Among 76 *S. aureus*, inducible macrolide–lincosamide–streptogramin B (MLS_B_) resistance, constitutive MLS_B_ resistance and macrolide–streptogramin B (MS_B_) resistance were seen in 17 (22.4%), 8 (10.5%) and 17 (22.4%) *S. aureus* respectively. Inducible MLS_B_ resistance was higher among MRSA in comparison to MSSA (p < 0.05) (Table 2).

**Table 2:** Inducible clindamycin resistance among strains of *S. aureus*

Resistant and susceptible phenotypes	Erythromycin	Clindamycin	D test	Total *S. aureus* (n = 76)	MRSA (n = 36)	MSSA (n = 40)
Inducible MLS_B_	R	S	+	17 (22.4%)	15 (41.7%)	2 (5%)
Constitutive MLS_B_	R	R	–	8 (10.5%)	4 (11.1%)	4 (10%)
MS_B_	R	S	–	17 (22.4%)	7 (19.4%)	10 (25%)
Susceptible	S	S	–	34 (44.7%)	10 (27.8%)	24 (60%)

*MLS*_*B*_ macrolide–lincosamide–streptogramin B, *MS*_*B*_ macrolide–streptogramin B

### Biofilm production among *S. aureus*

Thirty five (46.1%) *S. aureus* were found to be biofilm producers. Out of which, 23 (65.7%) were MRSA and 24 (68.6%) were MDR. Among 41 (53.9%) biofilm non producers, 13 (31.7%) were MRSA and 21 (51.2%) were MDR.

## Discussion

Similar rates of MRSA as in our study were reported by Shrestha et al. (44.9%) [12] and Ansari et al. (43.1%) [13]. In our study, higher percent of MRSA isolates were isolated from admitted patients. The colonized health care workers in the hospitals are the main sources of MRSA in hospitalized patients causing higher rates of infections among them [14]. However, the rate of isolation of MRSA among the out patients was also very high (41.9%). This high rate of community acquired MRSA infections indicates a frightening situation. The main source for the community acquired MRSA infections may be the colonized health care workers who transfer the MRSA to their household, spreading it to the community [14]. Further, the admitted patients who got colonized during hospital stay may also act as the alternative sources for the community acquired MRSA infections.

In our study, the rate of inducible clindamycin resistance was found to be 22.4%, which was very high in comparison to the rates reported in previous studies by Ansari et al. (12.4%) [13] and Adhikari et al. (10%) [15] in Nepal. Such difference could be due to the difference in resistance patterns of *S. aureus* in different patient groups, hospitals, time periods and geographical locations [12]. This showed the increasing prevalence of inducible clindamycin resistance among the clinical isolates of *S. aureus* in Nepal. We reported higher rate of inducible clindamycin resistance among the strains of MRSA in comparison to the strains of MSSA, which was in accordance to the result reported by Shrestha et al. [12]. Clindamycin is considered as one of the drugs of choice for treatment of the infection caused by MRSA [13]. But due to increasing rates of inducible clindamycin resistance among strains of *S. aureus* mainly MRSA, there is high chance of treatment failure if clindamycin is used for the treatment of the infections caused by strains showing inducible clindamycin resistance [1]. So, a simple test like D test for detection of inducible clindamycin resistance is crucial to guide the treatment of the infections caused by *S. aureus* and is recommended to include in the routine laboratory tests.

As in a study by Ansari et al. (94.7%) [13], high rate of resistance to penicillin (97.4%) was reported in our study. This is because only a small numbers of strains of *S. aureus* do not produce beta-lactamases [13]. We reported similar rate of resistance to ciprofloxacin (72.4%) as reported by Ansari et al. (63.7%) [13]. However, lower rate of resistance was reported toward gentamicin (31.6%) in comparison to the rate reported by Ansari et al. (60.4%) [13]. Further, higher rate of resistance to erythromycin (55.3%) was noted in contrast to 32.7% in a study by Ansari et al. [13]. In addition, resistance to cotrimoxazole was 63.2% in comparison to 81.7% noted in a previous study by Ansari et al. [13]. As previous studies in Nepal, no resistance was seen toward vancomycin and linezolid [13]. But among the commonly used antibiotics, the highest rate of susceptibility of *S. aureus* including MRSA, was found toward tetracycline followed by chloramphenicol suggesting the possibility of using these drugs for preliminary treatment of the infections caused by *S. aureus* in our settings. In the developing countries like Nepal, the low cost of these drugs will be an extra benefit.

In our study, higher rates of multidrug resistance and methicillin resistance were found among biofilm producing strains in comparison to biofilm non producing strains. These findings were in favor of the results reported by Ghasemian et al. [16]. Due to protective nature of the biofilm, the bacteria growing in it are intrinsically resistant to many antibiotics [17]. The antibiotic resistance among the strains of the bacteria residing in biofilm may increase up to 1000 times [17]. The main reasons for this may be difficulty in penetration of biofilm by antibiotics, slow growth rate of the bacteria and presence of antibiotic degradation mechanisms [17]. Further, biofilm formation gives platform for horizontal gene transfer among bacteria, causing the spread of drug resistance markers and other virulence factors [18].

In this study, due to lack of resources we could not use molecular methods to confirm our results but there are molecular methods like coagulase (coa) gene detection by polymerase chain reaction for identification of *S. aureus* [19] and detection of mecA gene for identification of methicillin resistant *S. aureus* [15].

## Conclusions

In our study, high rates of drug resistance among the strains of *S. aureus* to commonly used drugs were observed. The rate of isolation of MRSA from community acquired infections was found to be high in Nepal. Inducible clindamycin resistance is presenting as serious problem to the management of infections caused by *S. aureus*, as its prevalence is increasing in Nepal. So, D test for the detection of inducible clindamycin resistance should be included in the routine laboratory diagnosis to guide the treatment. Further, keeping in mind the high rate of biofilm production among the strains of *S. aureus* and high rate of drug resistance among the biofilm producing strains, detection of biofilm formation should also be included in the routine tests. On the basis of the antimicrobial susceptibility testing report of our study, among the commonly used drugs tetracycline and chloramphenicol can be used for the preliminary treatment of the infections caused by *S. aureus* including MRSA in our settings. However, we recommend to use linezolid and vancomycin for the preliminary treatment of the serious infections caused by *S. aureus*.

## Abbreviations

MRSA: methicillin resistant *S. aureus*
MDR: multidrug resistant
MSSA: methicillin susceptible *S. aureus*
MLS_B_: macrolide–lincosamide–streptogramin B
ATCC: American Type Culture Collection
SPSS: statistical package for the social sciences
MS_B_: macrolide–streptogramin B

## References

[1] Prabhu K, Rao S, Rao V (2011). Inducible clindamycin resistance in *Staphylococcus aureus* isolated from clinical samples. J Lab Physicians.

[2] Deotale V, Mendiratta DK, Raut U, Narang P (2010). Inducible clindamycin resistance in *Staphylococcus aureus* isolated from clinical samples. Indian J Med Microbiol.

[3] Lim HS, Lee H, Roh KH, Yum JH, Yong D, Lee K (2006). Prevalence of inducible clindamycin resistance in staphylococcal isolates at Korean Tertiary Care Hospital. Yonsei Med J.

[4] Steward CD, Raney PM, Morrell AK, Williams PP, McDougal LK, Jevitt L (2005). Testing for induction of clindamycin resistance in erythromycin resistant isolates of *Staphylococcus aureus*. J Clin Microbiol.

[5] Croes S, Deurenberg RH, Boumans ML, Beisser PS, Neef C, Stobberingh EE (2009). *Staphylococcus aureus* Biofilm formation at the physiologic glucose concentration depends on the *S. aureus* Lineage. BMC Microbiol.

[6] Cheesbrough M (2006). District laboratory practice in tropical countries, part II.

[7] Holt JG, Krieg NR, Sneath PHA, Staley JT, Williams ST (1994). Bergey’s manual of determinative bacteriology.

[8] Clinical and Laboratory Standards Institute. CLSI Document M100-S25. Performance standards for antimicrobial susceptibility testing: twenty fifth informational supplement edition. Wayne: CLSI; 2015.

[9] Magiorakos AP, Srinivasan A, Carey RB, Carmeli Y, Falagas ME, Giske CG (2012). Multidrug-resistant, extensively drug-resistant and pandrug-resistant bacteria: an international expert proposal for interim standard definitions for acquired resistance. Clin Microbiol Infect.

[10] Andrews JM (2001). Determination of minimum inhibitory concentrations. J Antimicrob Chemother.

[11] Los R, Sawicki R, Juda M, Stankevic M, Rybojad P, Sawicki M (2010). A comparative analysis of phenotypic and genotypic methods for the determination of the biofilm-forming abilities of *Staphylococcus epidermidis*. FEMS Microbiol Lett.

[12] Shrestha B, Pokhrel BM, Mohapatra TM (2009). Phenotypic characterization of nosocomial isolates of *Staphylococcus aureus* with reference to MRSA. J Infect Dev Ctries.

[13] Ansari S, Nepal HP, Gautam R, Rayamajhi N, Shrestha S, Upadhyay G (2014). Threat of drug resistant *Staphylococcus aureus* to health in Nepal. BMC Infect Dis.

[14] Pant ND, Sharma M (2016). Carriage of methicillin resistant *Staphylococcus aureus* and awareness of infection control among health care workers working in Intensive Care Unit of a Hospital in Nepal. Braz J Infect Dis.

[15] Adhikari R, Pant ND, Neupane S, Neupane M, Bhattarai R, Bhatta S (2017). Detection of methicillin resistant *Staphylococcus aureus* and determination of minimum inhibitory concentration of vancomycin for *Staphylococcus aureus* isolated from pus/wound swab samples of the patients attending a Tertiary Care Hospital in Kathmandu Nepal. Can J Infect Dis Med Microbiol.

[16] Ghasemian A, Peerayeh SN, Bakhshi B, Mirzaee M (2015). Several virulence factors of multidrug resistant *Staphylococcus aureus* isolates from hospitalized patients in Tehran. Int J Enteric Pathog.

[17] Neupane S, Pant ND, Khatiwada S, Chaudhary R, Banjara MR (2016). Correlation between biofilm formation and resistance toward different commonly used antibiotics along with extended spectrum beta lactamase production in Uropathogenic *Escherichia coli* isolated from the patients suspected of urinary tract infections visiting Shree Birendra Hospital, Chhauni, Kathmandu Nepal. Antimicrob Resist Infect Control.

[18] Soto SM (2014). Importance of biofilms in urinary tract infections: new therapeutic approaches. Adv Biol.

[19] Tiwari HK, Sapkota D, Sen MR (2008). Evaluation of different tests for detection of *Staphylococcus aureus* using Coagulase (coa) gene PCR as the gold standard. Nepal Med Coll J.

