# Erratum to: Antibiotic resistance and biofilm production among the strains of *Staphylococcus aureus* isolated in a tertiary care hospital in Nepal

**DOI:** 10.1186/s12941-017-0205-1

**Published:** 2017-04-13

**Authors:** Ankit Belbase, Narayan Dutt Pant, Krishus Nepal, Bibhusan Neupane, Rikesh Baidhya, Reena Baidya, Binod Lekhak

**Affiliations:** 1 Department of Microbiology, GoldenGate International College, Battisputali, Kathmandu, Nepal; 2 Department of Microbiology, Grande International Hospital, Dhapasi, Kathmandu, Nepal; 3 Department of Pathology, B&B Hospital, Gwarko, Lalitpur, Nepal; 4 Central Department of Microbiology, Tribhuvan University, Kirtipur, Nepal

## Erratum to: Ann Clin Microbiol Antimicrob (2017) 16:15 DOI 10.1186/s12941-017-0194-0

The authors of this paper [1] would like to include some extra information regarding the methodology of this research. This information is provided below:

- In the study, 830 various non-repeated clinical specimens were processed among which 76 (67 wound swab/pus samples, 5 catheter tips, 3 sterile body fluids and 1 tissue abscess) samples showed growth of *S. aureus*.

Appropriate changes to the text are listed below:

In the methods section of the abstract “830 various non-repeated clinical specimens” should replace “830 non-repeated pus/wound swab samples”.

In the methods section “A total of 830 various clinical samples” should replace “A total of 830 pus/wound swab samples”.

In the results section “Among total 830 various clinical samples processed” should replace “Among total 830 pus/wound swab samples processed”.

In the results section “Out of which, *S. aureus* was isolated from 76 (20.9%) samples (67 wound swab/pus samples, 5 catheter tips, 3 sterile body fluids and 1 tissue abscess)” should replace “Out of which, *S. aureus* was isolated from 76 (20.9%) samples”.

The author has also proposed a revised title for the paper: “Antibiotic resistance and biofilm production among the strains of *Staphylococcus aureus* isolated in a tertiary care hospital in Nepal” which has been included as the title of this erratum.
